# Structural Determinants Responsible for the Preferential Insertion of Ribonucleotides by Bacterial NHEJ PolDom

**DOI:** 10.3390/biom10020203

**Published:** 2020-01-30

**Authors:** Alejandro Sánchez-Salvador, Miguel de Vega

**Affiliations:** Centro de Biología Molecular Severo Ochoa (Consejo Superior de Investigaciones Científicas-Universidad Autónoma de Madrid), Nicolás Cabrera, 1, 28049 Madrid, Spain; asansal@outlook.es

**Keywords:** nonhomologous end joining (NHEJ), archaea/eukaryotic primase, DNA repair, ligase D

## Abstract

The catalytic active site of the Polymerization Domain (PolDom) of bacterial Ligase D is designed to promote realignments of the primer and template strands and extend mispaired 3′ ends. These features, together with the preferred use of ribonucleotides (*NTPs*) over deoxynucleotides (dNTPs), allow PolDom to perform efficient double strand break repair by nonhomologous end joining when only a copy of the chromosome is present and the intracellular pool of dNTPs is depleted. Here, we evaluate (i) the role of conserved histidine and serine/threonine residues in *NTP* insertion, and (ii) the importance in the polymerization reaction of a conserved lysine residue that interacts with the templating nucleotide. To that extent, we have analyzed the biochemical properties of variants at the corresponding His651, Ser768, and Lys606 of *Pseudomonas aeruginosa* PolDom (*Pa*-PolDom). The results show that preferential insertion of *NMPs* is principally due to the histidine that also contributes to the plasticity of the active site to misinsert nucleotides. Additionally, *Pa*-PolDom Lys606 stabilizes primer dislocations. Finally, we show that the active site of PolDom allows the efficient use of 7,8-dihydro-8-oxo-riboguanosine triphosphate (8oxo*GTP*) as substrate, a major nucleotide lesion that results from oxidative stress, inserting with the same efficiency both the *anti* and *syn* conformations of 8oxo*GMP*.

## 1. Introduction

DNA double-strand breaks (DSBs) are the most hazardous DNA lesions that have to be repaired in a timely fashion to prevent genome instability [1]. The two major DNA repair pathways to mend DSBs are homologous recombination (HR) and nonhomologous end-joining (NHEJ) (reviewed in [2]). Whereas HR relies on the presence of an intact chromatid copy used as the template for DNA synthesis across the break, NHEJ performs the direct ligation of the breaks, without needing a template strand. In the latter case, as the termini to be joined are often either non-compatible or are damaged, they are processed before the final ligation step, giving rise to insertions, deletions, and base substitutions at the break site, NHEJ being considered an error-prone pathway [2,3].

NHEJ is the main DSB repair mechanism that operates during the G1 phase of the eukaryotic cell cycle [4,5]. Briefly, the open ring-shaped Ku70/80 heterodimer binds the DNA ends by threading them through the internal ring [3,6,7]. Once bound to the DNA, Ku interacts with the DNA-dependent protein kinase catalytic subunit [8,9], and the resulting complex brings the break termini closer. Finally, Ku recruits the complex ligase IV/XRCC4/XLF/PAXX that joins the ends together [3,6,7]. Before the ligation step, non-compatible termini, as well as those containing lesions, are remodeled by the action of nucleases (Artemis and APLF) and of the family X DNA polymerases Pol λ, Pol μ, and TdT [3].

*In silico* analyses allowed the identification of eukaryotic Ku homologs in bacteria that led to the discovery of a two component NHEJ pathway in these organisms [10,11,12,13,14,15,16]. Genes encoding bacterial Ku are often co-operonic, with genes encoding an ATP-dependent DNA ligase (ligase D, LigD) [10,11,17,18]. This minimal NHEJ system is active and responsible for repairing DSBs that arise during the stationary and sporulation phases of the cell cycle where only a copy of the genome is available [13,19,20]. Whereas, in most cases, LigD is a multifunctional protein where the ligase domain (LigDom) is fused to a phosphoesterase (PEDom) and/or a polymerization (PolDom) domain responsible for processing incompatible termini, in several bacteria those domains exist as stand-alone proteins (reviewed in [21]). Extensive biochemical and structural characterization of the PolDom of *Mycobacterium tuberculosis* LigD (*Mt*-PolDom) allowed to decipher how NHEJ operates in bacteria to repair DSBs containing 3′-protruding ends [22,23,24]. Initially, Ku binds to both sides of the DSB and recruits LigD whose PolDom recognizes specifically the recessive 5′-P termini, forming a preternary precatalytic complex with Mn^2+^ ions and the incoming nucleotide that forms a Watson–Crick base pair with the templating nucleotide nearest the 5′-P end [23]. PolDom mediates further synapsis between the 3′ overhanging strands from opposing breaks. Non-extendable or damaged 3′ termini coming from the opposite break would be processed by the PEDom and the resulting 3′-OH end extended by the *in trans* addition of the nucleotide previously bound at the catalytic site of PolDom. The resulting nicks are finally sealed by the LigDom, fulfilling the break repair [24]. Although the bacterial NHEJ was originally envisaged as a minimal two components system, recent studies have brought to light the complexity of this repair pathway, due to the existence in some bacteria of several orthologues of Ku and/or of domains of LigD ([21] and references therein), as well as to the physical/functional interaction of other proteins with Ku and/or LigD [25,26].

Bacterial PolDom belongs to the archaeo-eukaryotic primase (AEP) superfamily distinguished by containing an RNA recognition motif [10,18,27,28]. This superfamily includes the RNA primases responsible for priming DNA replication in archaea and eukaryotes [28,29], as well as proteins endowed with both DNA primase and DNA polymerase activities (PrimPols), as the replication protein of plasmid pRN1 from *Sulfolobus islandicus* [30,31], and human PrimPol [32], whose DNA primase activity is essential to reinitiate stalled replication forks [29,33,34,35,36,37,38]. Structural and functional studies have revealed that bacterial NHEJ PolDom has an open active site that endows the protein with the flexibility required to bypass lesions, promote realignments of the template and primer strands during elongation of mispaired 3′ ends, and to add nucleotides in a template-independent manner (terminal transferase activity) on ssDNA and blunt-ended dsDNA substrates, facilitating the repair of DSBs [12,39].

The polymerization activity of bacterial PolDom in vitro is optimal in the presence of Mn^2+^ ions, ribonucleoside triphosphates (*NTPs*) being preferred over deoxynucleoside triphosphates (dNTPs) as substrates [12,39,40]. Such a preference might be advantageous when intracellular pools of dNTPs are depleted, as it occurs during the stationary phase where the presence of a single copy of the chromosome prevents DSBs repair by HR [41]. In this sense, it is worth mentioning that LigDom preferentially seals breaks with a 3′-OH monoribonucleotide [42].

In this work, we analyze the biochemical role in nucleotide insertion of invariant histidine and serine/threonine residues at the catalytic site of bacterial PolDom that interact with the 2′-OH group of the incoming *NTP*. Our results demonstrate that the preferential insertion of *NMPs* by PolDom is due mainly to the histidine residue that also contributes to the plasticity of the active site to misinsert *NMPs*. In addition, the biochemical analysis of site directed mutants at a lysine highly conserved among bacterial PolDoms allows us to propose a role for this residue in the dislocation of proximal templating nucleotides. Finally, we show that the active site of PolDom is flexible enough to accommodate both the *anti* and *syn* conformations of the incoming 8oxo*GTP* during an in vitro polymerization reaction.

## 2. Materials and Methods

### 2.1. Reagents and Oligonucleotides

Ultrapure dNTPs and *NTPs* were purchased from GE Healthcare (Buckinghamshire, UK). Ultrapure 8oxo*GTP* was purchased from TriLink (San Diego, CA, USA), lysozyme was from Sigma (San Luis, MO, USA), Benzonase was from Santa Cruz Biotechnology (Dallas, TX, USA). Oligonucleotides Cy5P (Cy5-5′-GATCACAGTGAGTAC); DowP (5′P-AACGACGGCCAGT); T29(X) (5′-ACTGGCCGTGCTTXGTACTCACTGTGATC; where X is dA, dC, dG or dT) and T30(AT) (5′-ACTGGCCGTGCTTTAGTACTCACTGTGATC) were from Integrated DNA Technologies (Coralville, IA, USA). The primer/template and gapped DNA substrates were obtained by hybridizing the primer to template and downstream oligonucleotides (1:1.2:1.2) in the presence of 60 mM Tris-HCl (pH 7.5) and 0.2 M NaCl and heating to 80 °C for 5 min before slowly cooling to room temperature overnight.

### 2.2. Expression and Purification of Recombinant Pa-PolDom

The region of *Pa*-LigD gene encoding PolDom (residues 533-840; [43]) was amplified by PCR using as substrate the recombinant plasmid pET16-*Pa*LigD [44], a sense strand primer that introduced an *Nde*I restriction site substituting the original codon for Arg532 by a methionine codon, and an antisense primer that introduced a *Bam*HI site. Both, plasmid pET16 and the PCR products were double digested with *Nde*I and *Bam*HI and further ligated to give rise to plasmid pET16-*Pa*-PolDom. The plasmid was transformed into *Escherichia coli* BL21(DE3) cells that were grown overnight in LB medium at 37 °C in the presence of (100 µg/mL) ampicillin. Cells were diluted into the same media and incubated at 30 °C until the A_600_ reached 0.6. Then, isopropyl β-d-thiogalactopyranoside (IPTG) was added to a final concentration of 1 mM and incubation was continued for 12 h at 15 °C. Cells were harvested by centrifugation and the pellets were stored at −80 °C. The slurry was resuspended in Buffer A (50 mM Tris-HCl, pH 7.5, 0.3 M NaCl, 7 mM β-mercaptoethanol, 5% glycerol) in the presence of 1 mg/mL of lysozyme and 25 U/mL of Benzonase, incubated for 30 min at 4 °C and further sonicated. The insoluble material was removed by centrifugation. The supernatant was loaded onto a Ni-NTA column (Qiagen; Venlo, The Netherlands) pre-equilibrated with Buffer A (0.3 M NaCl, 10 mM imidazole). The bound protein was eluted with 100–150 mM imidazole in Buffer A (0.1 M NaCl). The eluted protein was loaded onto a Heparin column (GE healthcare) pre-equilibrated with Buffer A (0.1 M NaCl). The bound protein was eluted with Buffer A (0.4 M NaCl) and further dialyzed against Buffer B (50 mM Tris-HCl, pH 7.5, 0.3 M NaCl, 7 mM β-mercaptoethanol, 50% glycerol, 0.05% Tween). The dialyzed protein was stored at −20 °C.

### 2.3. Site-Directed Mutagenesis

*Pa*-PolDom mutants K606R, K606A, H651A, S768Y, and S768A were obtained with the Q5^®^ Site-Directed Mutagenesis kit from New England Biolabs (Ipswich, MA, USA), following the manufacturer instructions and using plasmid pET16-*Pa*-PolDom as template for mutagenesis. Expression and purification of the mutant proteins were performed essentially as described for the wild-type PolDom.

### 2.4. Nucleotide Insertion Assays on Defined DNA Molecules

DNA-dependent nucleotide insertion was assayed on a primer/template substrate [obtained by hybridization of oligonucleotides Cy5P and T29(X)], on 1-nt gapped molecules (obtained by hybridization of oligonucleotides Cy5P, T29(X), and DowP, which contains a 5′-phosphate) and on 2-nt gapped molecules (obtained by hybridization of oligonucleotides Cy5P, T(30)AT and DowP). The incubation mixture (12.5 μL) contained 50 mM Tris-HCl pH 7.5, 5 mM MnCl_2_, 1 mM dithiothreitol (DTT), 4% (*v/v*) glycerol, 0.1 mg/mL bovine serum albumin (BSA), and the indicated concentrations of the specified hybrid, nucleotide and enzyme. After incubation at 30 °C for the indicated times, the reactions were stopped by adding ethylendiaminetetraacetic acid (EDTA) to 10 mM. Samples were analyzed by 7 M urea-20% polyacrylamide gel electrophoresis (PAGE) and visualized using a Typhoon 9410 scanner (GE Healthcare).

### 2.5. Steady-State Primer Extension Assays

Steady-state experiments were performed essentially as described [45]. Thus, pre-hybridized Cy5P and T29(A) (1:1.2) were mixed with either wild-type, S768Y, S768A, or H651A *Pa*-PolDom. Reaction times and enzyme concentrations were adjusted in each case to optimize the product detection, while ensuring that all reactions were conducted in the steady state. Only those reactions that fell within the linear range of substrate utilization (≤25% primer extension) were used for analysis. *Steady-state primer extension assays with UTP*. The mixture contained (in 11.5 μL) 50 mM Tris-HCl, pH 7.5, 5 mM MnCl_2_, 1 mM DTT, 4% (*v/v*) glycerol, 0.1 mg/mL BSA, 200 nM of the DNA substrate, and either wild-type (2 nM), S768Y (4 nM), S768A (2 nM), or H651A (2 nM). The protein/DNA mixture was directly added to varying concentrations (0–800 nM) of *UTP* to start the polymerization reaction. Samples were incubated at 30 °C for either 1.3 min (wild-type, S768Y and S768A) or 5 min (H651A). *Steady-state primer extension assays with dTTP*. The assay was carried out as described above in the presence of either 4 nM (wild-type, S768A and H651A) or 32 nM (S768Y) *Pa*-PolDom. Samples were incubated at 30 °C for 5 min. *Steady-state primer extension assays with GTP*. The assay was carried out as described above in the presence of either 8 nM (wild-type and S768Y), 32 nM (S768A), or 64 nM (H651A) *Pa*-PolDom. Samples were incubated at 30 °C for either 7 min (wild-type and S768A), 14 min (S768Y), or 30 min (H651A). In all cases, after incubation for the indicated times, reaction mixtures (12.5 µL) were quenched by the addition of 10 µL of 95% (*v/v*) formamide, 20 mM EDTA. Extension of the Cy5-5′-labeled primer strand was analyzed by 7M urea and 20% PAGE and visualized using a Typhoon 9410 scanner (GE Healthcare). Gel band intensities were quantified using Image Lab software (6.0.1, Bio-Rad Laboratories, Hercules, CA, USA). The observed rate of nucleotide incorporation (extended primer) was plotted as a function of nucleotide concentration. Steady-state kinetic parameters, V_max_ and *K_m_*, were determined by fitting the data to the Michaelis–Menten equation:

V = V_max_[S]/(*K_m_* + [S]), (1)

*k_cat_* was determined with the equation:

*k_cat_* = V_max_/[E], (2)

### 2.6. Electrophoretic Mobility Shift Assays (EMSAs)

EMSAs were used to analyze the interaction of *Pa*-PolDom with a 1 nt gapped substrate in a final volume of 12.5 µL, containing 50 mM Tris-HCl, pH 7.5, 1 mM DTT, 4% (*v/v*) glycerol, 0.1 mg/mL BSA, 50 nM Cy5-5′-labeled DNA, and the indicated concentrations of either the wild-type or mutant *Pa*-PolDom. After incubation for 10 min at 4 °C, samples were mixed with 3 µL of 30% glycerol and resolved by native gel electrophoresis on a 6% polyacrylamide gel. After electrophoresis, the binary complex protein/DNA and free DNA were detected using a Typhoon 9410 scanner (GE Healthcare).

### 2.7. Kinetic Measurement of 8oxoGMP Incorporation by *Pa*PolDom

The mixture contained (in 150 µL) 50 mM Tris–HCl, pH 7.5, 5 mM MnCl_2_, 1 mM DTT, 4% (*v/v*) glycerol, 0.1 mg/mL BSA, 5 nM of the indicated gapped DNA molecule, and 40 nM of either 8oxo*GTP*, *GTP*, or *UTP*. Reactions were initiated by adding 20 nM *Pa*PolDom and incubated at 30 °C. Aliquots (12.5 µL each) were withdrawn at different time intervals and stopped with 10 mM EDTA. The reaction products were analyzed by 7 M urea-20% PAGE and visualized using a Typhoon 9410 scanner (GE Healthcare). Reaction rates (*k*_obs_ in s^−1^) of 8oxo*GMP* incorporation opposite a template dC and dA, as well as *UMP* insertion opposite dA and *GMP* insertion opposite dC, were determined by plotting the percentage of the elongated primer as a function of time, and data were fitted to an exponential equation by least-squares nonlinear regression.

## 3. Results and Discussion

### 3.1. Role of *Pa*-LigD Residues His651 and Ser768 in Preferential Insertion of Ribonucleotides and Fidelity

**Figure 1:**
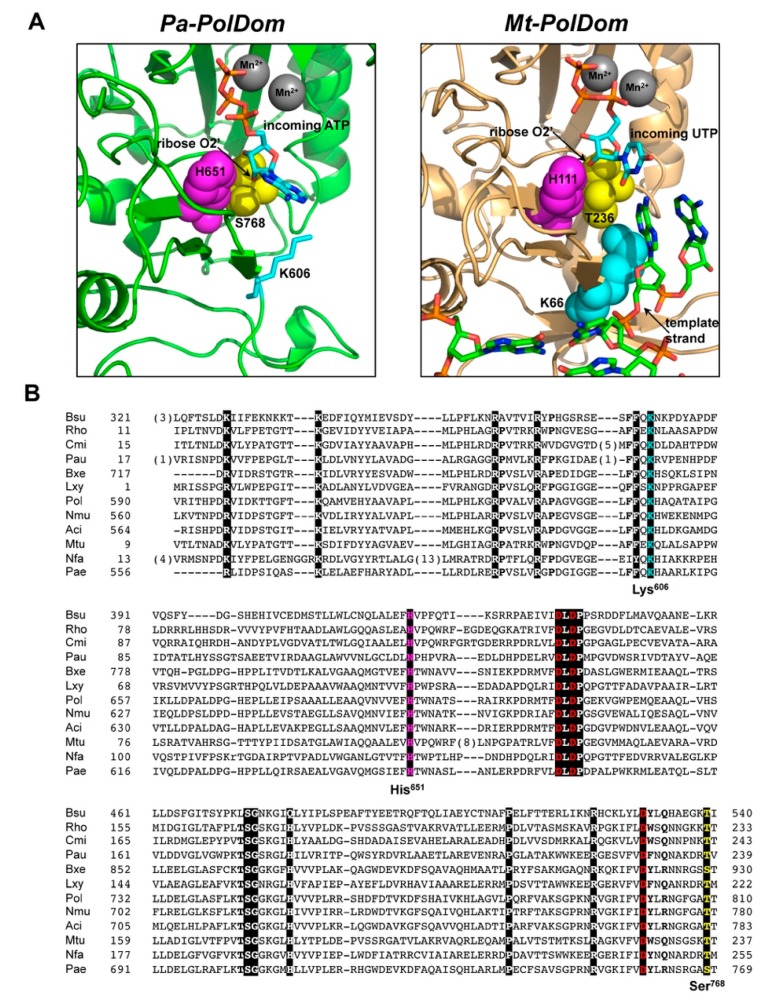
Catalytic active site of bacterial PolDom. (**A**) Detailed view of the incoming *NTP* insertion site of *Pa*-PolDom (PDB 2FAQ; [31]; left panel) and *Mt*-PolDom (PDB 3PKY; [23]; right panel). 2′-OH contacting residues His651 and Ser768 (*Pa*-LigD) and the homologs His111 and Thr236 (*Mt*-LigD) studied here are represented as magenta and yellow spheres. Template strand interacting residue Lys66 (*Mt*-LigD) and the homolog Lys606 (*Pa*-LigD) is shown as blue spheres. (**B**) Multiple amino acid sequence alignment of the PolDom of bacterial LigDs. Numbers indicate the amino acid position relative to the N-terminus of each LigD. Because of the large number of sequences, only selected representatives are aligned. Name of organisms are abbreviated as follows: Bsu, *Bacillus subtilis*; Rho, *Rhodococcus*; Cmi, *Clavibacter michiganenses*; Aau, *Arthobacter aurescenses*; Bxe, *Burkholderia xenovorans*; Lxy, *Leifsonia xyli*; Pol, *Polaromonas*; Nmu, *Nitrosospira multiformis*; Aci, *Acidovorax*; Pae, *Pseudomonas aeruginosa*; Mtu, *Mycobacterium tuberculosis*; Nfa, *Nocardia farcinica*. Catalytic aspartates are indicated in red letters, residues homologous to *Pa*-LigD His651 and Ser768 in magenta and yellow, respectively, and conserved lysine corresponding to *Pa*-LigD Lys606 is indicated in cyan. Other highly conserved residues are indicated with white letters over a dark background.

The crystallographic structures of the PolDom of *P. aeruginosa* LigD (*Pa-*PolDom) with a Mn^2+^-*ATP* bound at the active site [31], as well as of the ternary complex of *Mt-*PolDom with an incoming *UTP* forming a Watson–Crick base pair with a templating adenine base [23] showed that the ribose 2′-OH group of the nucleotide is hydrogen bonded to the Nδ moiety of a conserved histidine residue (*Pa-*LigD His651, *Mt-*LigD His111; see Figure 1). In addition, the structures of *Pa-*PolDom-*ATP* and *Mt-*PolDom ternary complex also disclosed a contact between the ribose 2′-OH group of the incoming nucleotide with the side-chain hydroxyl group of *Pa-*LigD Ser768 [31] and the homologous Thr236 of *Mt-*LigD [23] (see Figure 1A,B). Therefore, these observations led us to analyze the role for those residues in the preferential insertion of *NMPs* exhibited by bacterial LigDs. To that purpose, we expressed and purified the PolDom of *Pa-*LigD (residues 533–840, [43]) as well as variants at the corresponding residues His651 (mutant H651A) and Ser768 (mutants S768A and S768Y), as described in Materials and Methods.

**Figure 2:**
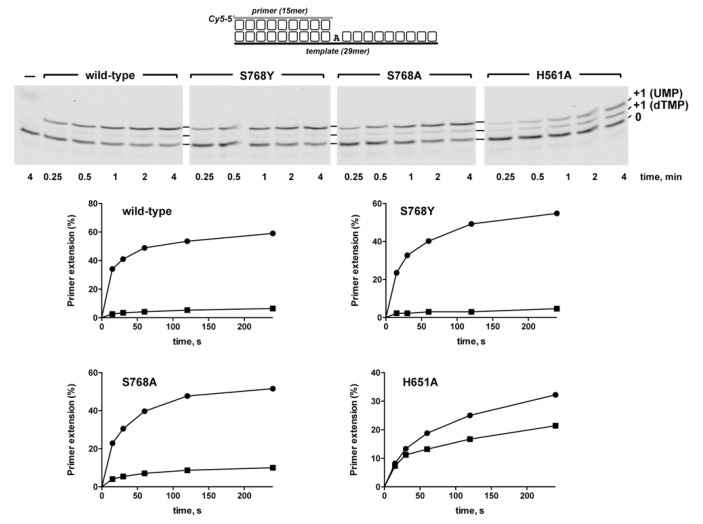
Sugar discrimination by *Pa*-PolDom wild-type and mutants S768Y, S768A, and H651A. Upper panel, primer extension assays were performed using 10 nM of the primer/template substrate depicted at top, 25 nM of either the wild-type or the specified *Pa*-PolDom mutant, and 100 nM of both dTTP and *UTP*. Samples were incubated at 30 °C for the indicated times, products were resolved by denaturing PAGE and further visualized using a Typhoon 9410 scanner (GE Healthcare). Lower panel, the extent of primer extension was quantified using the ImageLab software (BioLabs), and the data plotted as a function of time. Full circles and full squares correspond to insertion of *UMP* and dTMP, respectively.

To ascertain qualitatively how the introduced changes affected the sugar selectivity by *Pa-*PolDom, we analyzed nucleotide insertion on the template/primer substrate depicted in Figure 2 where the first templating nucleotide is dAMP, providing simultaneously both sugars (*UTP* and dTTP), as described in [46,47]. Due to the different molecular weights of *UTP* and dTTP, the +1 extended primers are separated by gel electrophoresis and further quantified to obtain the sugar selectivity factor [*S* = % (primer extension with *UTP*)/% (primer extension with dTTP)] [47]. As expected, the wild-type enzyme showed a marked preference for *UMP* insertion (*S* = 7; see Figure 2). The selectivity factor displayed by mutants S768Y and S768A was similar to that of the wild-type enzyme (9 and 5, respectively). In contrast, the discrimination against deoxynucleotide insertion dropped drastically in mutant H651A (*S* = 1.4) supporting the involvement specifically of this residue in the preferential ribonucleotide usage exhibited by these enzymes.

**Figure 3:**
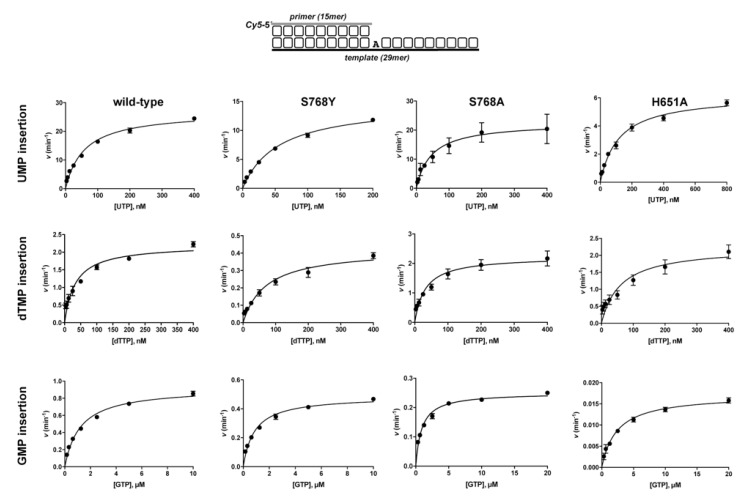
Nucleotide incorporation by *Pa*-PolDom wild-type and mutants S768Y, S768A, and H651A. The assays were performed under steady-state conditions as described in Materials and Methods. The turnover values (*v* in min^−1^) of nucleotide incorporation opposite a template dA were plotted as a function of nucleotide concentration and fitted to the Michaelis–Menten equation by least-squares nonlinear regression. The *k_cat_*, *K_m_* and catalytic efficiency (*k_cat_*/*K_m_*) values are given in Table 1.

**Figure 4:**
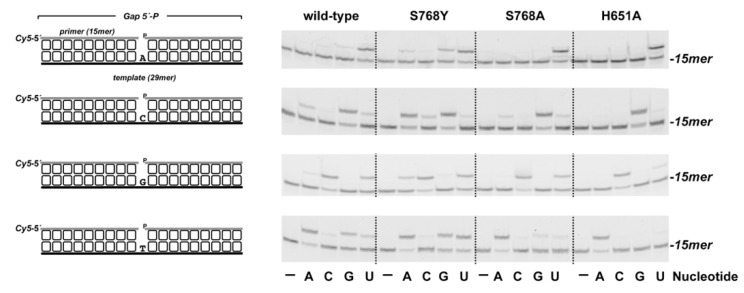
Nucleotide insertion fidelity by *Pa*-PolDom wild-type and mutants S768Y, S768A, and H651A at 1 nt gapped DNA. The four different 1 nt gapped structures used, differing in the templating base are shown on the left. The assay was performed as described in Materials and Methods with 10 nM of the indicated substrate, 5 nM of either the wild-type or the specified *Pa*-PolDom mutant and 50 nM of the indicated *NTP*. Samples were incubated at 30 °C for 3 min, products resolved by denaturing PAGE and further visualized using a Typhoon 9410 scanner (GE Healthcare).

**Figure A1:**
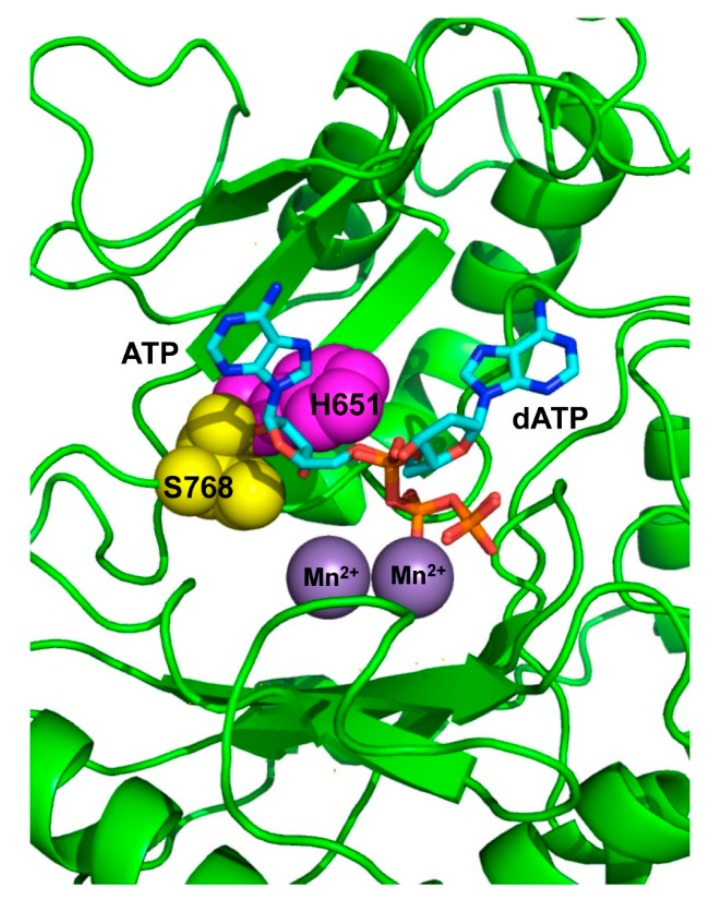
Detailed view of the conformations adopted by ATP and dATP at the catalytic site of *Pa*-PolDom. Figure was done by overlapping the structures of the *Pa*-PolDom/ATP (PDB 2FAQ; [31]) and *Pa*-PolDom/dATP (PDB 2FAQ; [31]) complexes. 2′-OH contacting residues His651 and Ser768 studied here are represented as magenta and yellow spheres. See main text for details.

To study in detail the *NTP*/dNTP insertion by the wild-type and mutant enzymes we determined their steady-state kinetic parameters [*k_cat_* and *K_m(app)_*], as well as the catalytic efficiencies [*k_cat_/K_m(app)_*] for *UMP* and dTMP incorporation. As shown in Figure 3 and Table 1, the wild-type *Pa-*PolDom displayed a catalytic efficiency for *UMP* incorporation 7-fold higher than for dTMP, the discrimination against dTMP relying principally on a low catalytic rate rather than on an increased *K_m_* for the nucleotide. As shown, the replacement of Ser768 by alanine had no impact on the incorporation of both *UMP* and dTMP, as the kinetic parameters obtained with mutant S768A were comparable to those exhibited by the wild-type enzyme. This result indicates that the interaction between the ribose 2′-OH group of the incoming nucleotide with the hydroxyl group of *Pa-*PolDom Ser768 is not essential, neither for nucleotide insertion, as previously described [31], nor for sugar discrimination. Interestingly, tyrosine substitution for Ser768 increased nucleotide discrimination against dTMP (*f* = 44, see Figure 4 and Table 1) because of a severe reduction of the *k_cat_* for dTMP insertion. The crystallographic structure of the *Pa-*PolDom forming a complex with dATP showed that the adenosine was folded back over the triphosphate moiety, unlike the extended conformation adopted in the *ATP*-complex [31] (see also Figure A1). Similarly, the comparison of the binary complexes of *Mt-*PolDom with *GTP* and dGTP showed that whereas the guanosine base of *GTP* was positioned away from the triphosphate tail, that of the dGTP adopted a number of orientations at the active site [48]. Those results led to surmise the existence of two nucleotide binding modes conditioned by the sugar moiety of the incoming nucleotide. If this were the case, the base of dNTPs should rotate from the non-catalytically competent conformation to the extended one to allow the proper pairing of the base with the templating nucleotide. Thus, it is tempting to speculate that the presence of the aromatic group in mutant S768Y could be hindering the base rotation in dTTP towards the productive conformation.

Regarding mutant H651A, alanine substitution for His651 impaired specifically *UMP* insertion (see Figure 3 and Table 1). Thus, whereas the catalytic efficiency for dTMP was only 1.8-fold lower than that of the wild-type enzyme, the change introduced in *Pa-*PolDom caused a 9-fold decrease in *UMP* insertion efficiency due to both a 2-fold increase of the *K_m_* and a 4-fold lessening of the catalytic rate, causing a nearly complete loss of discrimination in the insertion of the two nucleotides in the PolDom variant. A recent comparative analysis of the structures of *Mt-*PolDom with those of two widely characterized AEP members, *Pyrococcus furiosus* p41(Pfu-p41), and human PrimPol, both exhibiting a markedly preference for dNMP insertion [32,49], showed a similar spatial arrangement among *Mt-*LigD His111 (the homolog residue to *Pa-*PolDom His651), *Pfu*-p41 Tyr72 and human PrimPol Tyr100 [50]. Importantly, biochemical analysis of PrimPol mutant Y100H stimulated *NMPs* insertion, a result that allowed authors to propose that the bulky side chain of Tyr100 would clash with the 2′-OH group of *NTPs* acting as the steric gate residue responsible for sugar discrimination in this protein [50]. These observations led authors to surmise that the residue located at the equivalent position would dictate the nucleotide sugar use, histidine and tyrosine favoring the incorporation of *NTP* and dNTPs, respectively [50]. The results shown here with *Pa-*PolDom mutant H651A confirm such a hypothesis. That *Pa-*PolDom mutant H651A exhibited the same catalytic efficiency for the incorporation of *UMP* and dTMP indicates that the discrimination against dNMPs incorporation in bacterial LigDs would be accomplished exclusively by the contact between the ribose 2′-OH group and the Nδ moiety of the histidine residue.

PolDom catalyzes nucleotide insertion in a very unfaithful manner [43,48,51]. This fact, together with both its template-independent terminal transferase activity on blunt-ended duplex DNAs and its ability to promote dislocations of the template and primer strands contribute to the high error rate of bacterial NHEJ [31,43,48,51,52,53]. Nucleotide insertion fidelity in “canonical” DNA polymerases resides in the induced-fit formation of a tight-binding pocket around the nascent base pair, and whose geometry allows the accommodation only of a correct Watson−Crick base pair [54]. The initial structures of (*Mt*/*Pa*)*-*PolDom-*NTP* binary complexes led the authors to propose that the PolDom infidelity was due to the low contacts between the incoming nitrogen base and the enzyme, initially restricted mainly to a stacking interaction between the base and the aromatic ring of a conserved Phe residue (*Pa/Mt−*LigD Phe604/Phe64) [31,48]. However, further crystallization of a *Mt−*PolDom preternary complex showed that in the presence of a template strand, Phe64 changes its overall conformation to stack against the templating base, no longer interacting with the base of the incoming nucleotide [23]. Considering that *Pa−*PolDom residues Ser768 and His651 interact with the 2′−OH group of the incoming *NTP*, we analyzed how the lack of such contacts in mutants Ser768A, Ser768Y, and His651A affect the nucleotide insertion fidelity of the enzyme. Thus, we selected the 1 nt gapped DNA substrates depicted in Figure 4 and tested the +1 extension of the labeled primer after adding each of the four *NTPs* individually, covering the 16 possible nucleotide pairs. As observed, although in all cases the template nucleotide directed the preferential addition of the complementary nucleotide, both the wild-type and mutant S768Y, and at a lower extent, S768A, showed a significant level of misincorporation. In contrast, mutant H651A discriminated against the insertion of erroneous nucleotides much more efficiently than the wild-type enzyme. The determination of the steady-state parameters for the misinsertion of *GMP* opposite dA (see Table 1) and their comparison with those previously obtained for the correct insertion of *UMP* (nucleotide insertion fidelity *F*_ins_: (*k*_cat_*/K*_m_)*_UTP_*/(*k*_cat_/*K*_m_)*_GTP_*) rendered a 2.8- and 11.8-fold higher fidelity in mutants S768A and H651A than in the wild-type PolDom (see Table 1), mainly due to a decrease in the catalytic rate for *GMP* insertion. This result could indicate that the contacts between the 2′-OH group of the incoming nucleotide and the protein would be pivotal, not only to allow preferential *NMP* insertion but also to accommodate/stabilize a mispaired nucleotide at the catalytic site to allow further phosphodiester bond formation with the in trans coming 3′-OH end. In this sense, the lack of a 2′-OH group makes PolDom much more faithful during the insertion of dNTPs [51]

### 3.2. Role of Pa-LigD Lys606 in Dislocation of Proximal Templating Nucleotides

The inspection of the ternary complex of *Mt-*PolDom shows the presence of a lysine residue (Lys66) that packs against the template nucleotide, maintaining its spatial orientation [22,23,24]; see also Figure 1. This lysine residue is absolutely conserved in all LigDs [[24], see also Figure 1B] and would correspond to *Pa-*LigD Lys606. Both, the spatial location and the conservation of such a lysine suggest a role for this residue during the nucleotide insertion reaction. To ascertain the functional importance of this lysine residue in bacterial LigDs, we introduced single changes at the corresponding *Pa-*PolDom residue Lys606, obtaining the derivatives K606R and K606A, further overproduced and purified as described in Materials and Methods.

**Figure 5:**
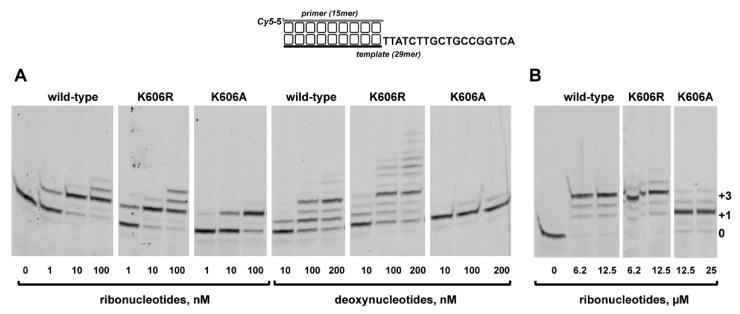
Nucleotide incorporation by *Pa*-PolDom wild-type and mutants K606R and K606A. The assays were performed as described in Materials and Methods, using 5 nM of the primer/template substrate depicted at top, 200 nM of either the wild-type or the specified *Pa*-PolDom mutant. The assay was performed in the presence of the indicated nanomolar (**A**) and micromolar (**B**) range of either dNTPs or *NTPs*. Samples were incubated at 30 °C for 5 min, products were resolved by denaturing PAGE and further visualized using a Typhoon 9410 scanner (GE Healthcare).

**Figure 6:**
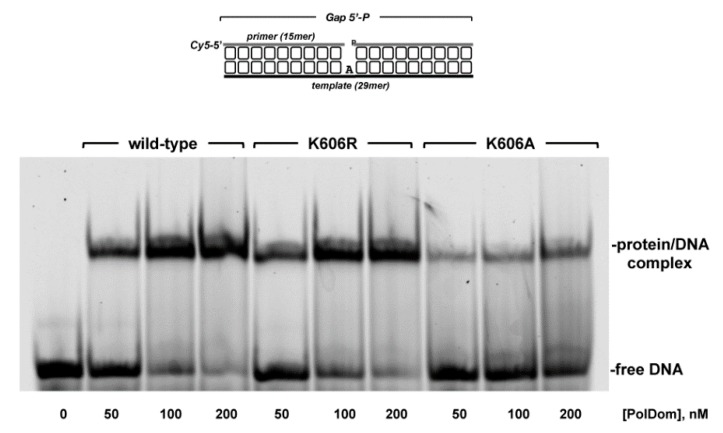
Gel retardation of a primer/template substrate by *Pa*-PolDom wild-type and mutants K606R and K606A. The assay was carried out as described in Materials and methods, using 50 nM of the depicted primer/template substrate, in the presence of the indicated amount of either wild-type or the indicated *Pa*-PolDom mutant. After gel electrophoresis, the bands corresponding to free DNA and to PolDom-DNA complexes were visualized using a Typhoon 9410 scanner (GE Healthcare).

**Figure 7:**
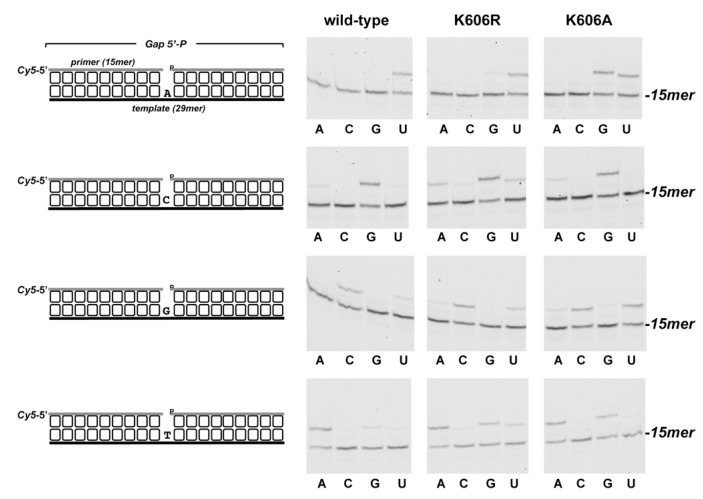
Nucleotide insertion fidelity by *Pa*-PolDom wild-type and mutants K606R and K606A at 1 nt gapped DNA. The four different 1 nt gapped structures used, differences in the templating base are shown on the left. The assay was performed as described in Materials and Methods with 10 nM of the indicated substrate, 5 nM of either the wild-type or the specified *Pa*-PolDom mutant, and 50 nM of the indicated *NTP*. Samples were incubated at 30 °C for 3 min, products were resolved by denaturing PAGE and further visualized using a Typhoon 9410 scanner (GE Healthcare).

As shown in Figure 5A, the wild-type and *Pa-*PolDom mutants catalyzed the template directed addition of both *NTP* and dNTP on a primer/template substrate, and in all cases insertion of *NTP* was more efficient than that of dNTP. Due to the strong preference by *Pa-*PolDom for a DNA primer terminus versus an RNA primer [55], both the wild-type and mutant K606R proteins inserted 3*−*4 ribonucleotides and up to 6 (wild-type) and 10 (K606R) deoxynucleotides. Interestingly, whereas mutant K606R was slightly more proficient than the wild-type enzyme in the insertion of both *NTP* (the +3/+2 product ratio obtained with mutant K606R was 3-fold higher than that obtained with the wild-type enzyme, Figure 5A) and dNTP, K606A mutant exhibited an overall low nucleotide incorporation efficiency, *NTP* insertion being mainly limited to the addition of one nucleotide (Figure 5A and B). Considering the potential interaction of residue Lys606 with the template strand, we tested the DNA binding ability of mutant proteins by an electrophoretic mobility shift assay (EMSA). As observed in Figure 6, mutant K606A was less efficient at binding to the substrate than the wild-type and K606R mutant. This result would explain the reduced polymerization efficiency exhibited by mutant K606A, agreeing with a DNA binding role for Lys606. Interestingly, despite the lower insertion activity displayed by mutant K606A, its relative nucleotide misincorporation efficiency was even higher than that of the wild-type enzyme (Figure 7). This result could indicate that the smaller size of the Ala side chain in mutant K606A increases the plasticity of the active site, allowing a better accommodation of mispairs and suggesting that the stabilization of the templating nucleotide by Lys606 would limit nucleotide misinsertion in bacterial PolDoms.

**Figure 8:**
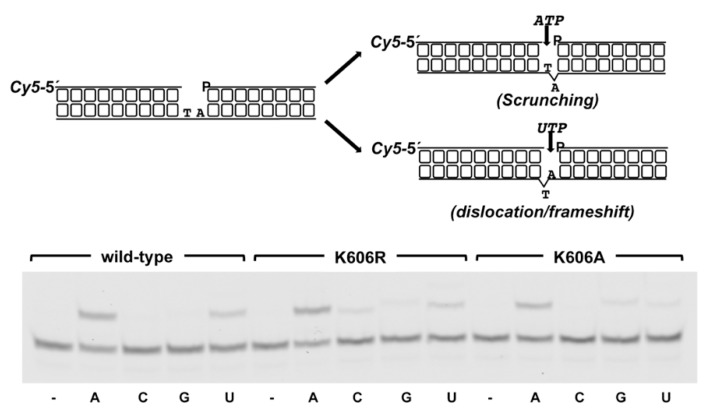
Selection of the templating base by *Pa*-PolDom wild-type and mutants K606R and K606A. Gap-filling reactions were performed as described in Materials and Methods using 5 nM of the depicted 2 nt gapped DNA substrate, 10 nM (wild-type and K606R) and 30 nM (K606A) *Pa*-PolDom and 10 nM of the indicated *NTP*. After incubation at 30 °C for 5 min, products were resolved by denaturing PAGE and further visualized using a Typhoon 9410 scanner (GE Healthcare).

As alluded to early, the structural and functional studies of *Mt-*PolDom led to envision how PolDom accounts for DSB repair during a NHEJ reaction [22,23]. PolDom recognizes the recessive 5′-P group of the DSB through the conserved 5′-P binding pocket. The templating nucleotide nearest to the 5′-P end directs the formation of a Watson−Crick base pair with the incoming nucleotide that will be further incorporated *in trans* onto the 3′*-*OH end of an incoming primer end [23]. Gapped molecules have been extensively used as NHEJ intermediates to analyze the biochemical properties of PolDoms. The studies carried out with the *Mt-*PolDom acting on a 2 nt gapped molecule allowed the observation that the first nucleotide directs insertion of the incoming nucleotide [48]. To reconcile this result with the previous finding, that in most cases the base closest to this 5′ terminus selects the incoming nucleotide [23], it was predicted that PolDom scrunches the second nucleotide of the gap. Thus, PolDom binds simultaneously the 3′ primer terminus and the 5′-P of the downstream strand and flips out the second nucleotide, keeping the same distance between both termini as in a 1 nt gapped substrate, allowing further nucleotide insertion, a mechanism that has been also described for the eukaryotic NHEJ Pol λ [56] (see also scheme in Figure 8). Alternatively, although to a lower extent, *Mt-*PolDom can use also the second nucleotide of the gap by dislocating one of the proximal upstream bases in the template strand (frameshift/dislocation mechanism) [22,48]. Thus, the ability of PolDom to promote different nucleotide distortions could be important for the NHEJ reaction when the protruding 3′ ends to be joined have a limited complementarity and the synapsis between them gives rise to gaps of more than a single nucleotide [22,48].

Therefore, to study whether those observations could be extended to other PolDoms, as well as to ascertain the role of the conserved lysine residue, we analyzed nucleotide incorporation in a 2 nt gapped DNA. As shown in Figure 8, as *Mt-*PolDom, *Pa-*PolDom inserts preferentially *AMP*, complementary to the first position of the gap (44% of elongated primer molecules) suggesting that it scrunches the second nucleotide. Insertion of *UMP* also takes place, although to a lower extent (23% of elongated primer molecules). Interestingly, whereas the nucleotide insertion behavior of mutant K606R is similar to that of the wild-type protein, the dislocation/frameshift ability of mutant K606A was seriously compromised, as it inserted *UMP* opposite the second nucleotide (7% of elongated primer molecules) ~5-fold less efficiently than *AMP* opposite the first one (33% of elongated molecules). This fact suggests that the conserved *Pa-*PolDom Lys606 would be a ligand important to stabilize/promote the dislocation of upstream bases in the template strand. Conversely, structural and biochemical studies carried out in *Mt-*PolDom have demonstrated that residues placed at the β-hairpin structure called loop 1, conserved in the PolDom of most bacterial LigDs and critical to maintain both the synapsis between the two DNA termini [24] and the orientation of the template strand, are responsible for promoting scrunching [22]. Altogether, the results presented here strongly support the notion that the plasticity of PolDom to choose between the two potential templating nucleotides would require the presence of template strand ligands involved in the stabilization of the scrunched and/or the dislocated nucleotide [22].

### 3.3. 8oxoGMP Is Efficiently Inserted During In Vitro Polymerization by PolDom

One of the main sources of genomic lesions are the reactive oxygen species (ROS) that are by-products of the cellular metabolism, and whose formation can be enhanced by the exposure to ionizing radiation and several genotoxicants [1,57,58,59]. ROS can oxidize purine and pyrimidine bases of the genomes and of the nucleotide pool, 8-oxo-7,8-dihydroguanine being the most abundant oxidized base. The toxicity of this lesion (8oxodGTP/*GTP* in the nucleotides pool, 8oxodGMP in the DNA) resides in its ability to form the 8oxoG:dC (*anti*/*anti*) and 8oxoG:dA (*syn*/*anti*) base pairs during nucleic acid synthesis whose persistence in the genomes can cause DNA replication and gene expression errors, as well as transversion mutations [60].

**Figure 9:**
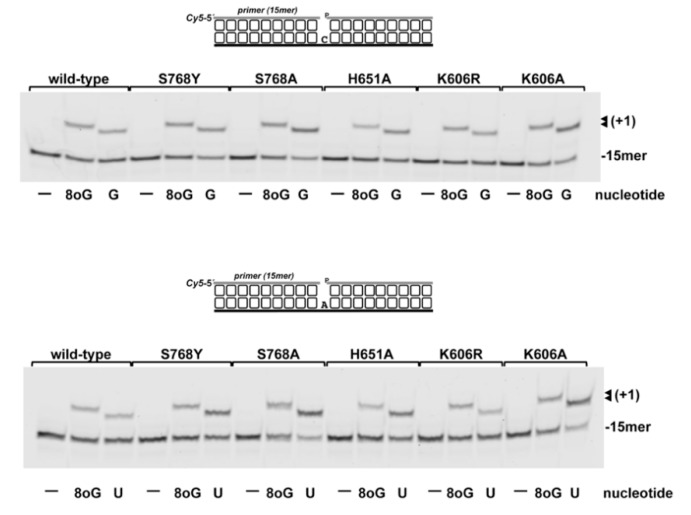
8oxo*GTP* insertion by *Pa*-PolDom wild-type and mutants. The assays were carried out as described in Materials and Methods, incubating 40 nM of the depicted 1 nt gapped DNA substrate with 5 nM of either the wild-type or the specified mutant *Pa*-PolDom (20 nM in the case of K606A mutant), and 40 nM of the indicated nucleotide (8oxo*GTP*, *UTP*, *GTP*). After incubation at 30 °C for 5 min, products were resolved by denaturing PAGE and further visualized using a Typhoon 9410 scanner (GE Healthcare).

**Figure 10:**
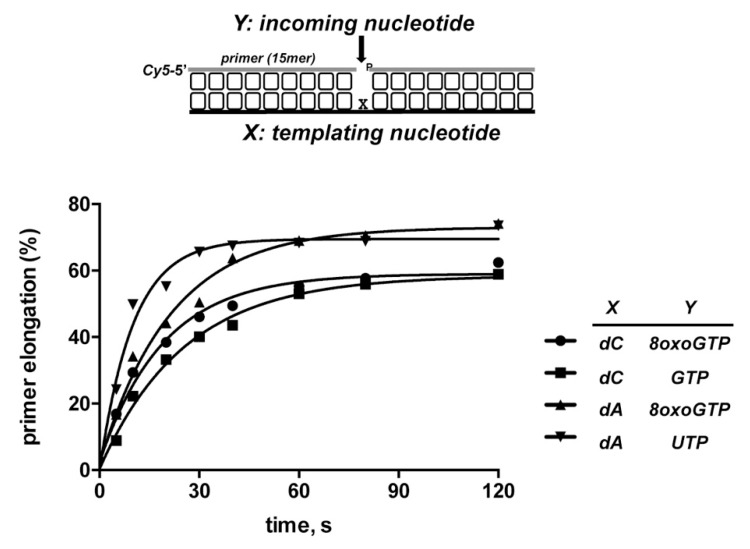
Kinetic measurement of 8oxoGMP incorporation by *Pa-*PolDom. The assay was performed as described in Materials and Methods. Insertion of the indicated nucleotide was examined as a function of the incubation time, and the rate of nucleotide incorporation was determined under single-turnover conditions by incubating 5 nM of the indicated gapped molecule (depicted in top of the figure), 40 nM of of either 8oxo*GTP*, *GTP* or *UTP* and 20 nM *Pa*-PolDom. Time course data were analyzed using a Typhoon 9410 scanner (GE Healthcare) and fitted to an exponential equation by using nonlinear least squares methods to determine the reaction rate (*k_obs_*).

Previous results showed the ability of PolDoms to carry out non-mutagenic translesion synthesis past 8oxodGMP, as the enzyme inserts *CMP* much more efficiently than *AMP*, and at a similar extent as opposite the non-damaged dGMP [48]. Considering both the preferential insertion of *NMP* by PolDom and that the oxidation of guanine also takes place in the ribonucleotides pool of cells, it was relevant to analyze the capacity of *Pa-*PolDom to use 8oxo*GTP* as the nucleotide during the insertion reaction. For this, purified PolDom was incubated with a 1nt gapped DNA substrate harboring either dC or dA as templating base (see Figure 9). As shown, the wild-type enzyme incorporated 8oxo*GMP* with a similar efficiency opposite dC or dA. The determination of the nucleotide insertion rate under single turnover conditions (i.e., [enzyme]/[DNA] = 4) rendered an observed rate (*k*_obs_) of ~0.05/sec in both cases (see Figure 10). Importantly, the non-damaged nucleotide *GMP* and 8oxo*GMP* were both inserted opposite dC with a similar efficiency (the *k*_obs_ for *GMP* insertion was ~0.04/sec), insertion opposite dA of *UMP* being slightly more efficient than that of 8oxo*GMP* (*k*_obs_ ~0.09/sec). As shown in Figure 9, none of the changes introduced in PolDom affected significantly the relative insertion of 8oxo*GMP*.

The structures of the DNA polymerases, where 8-oxo-7,8-dihydroguanine is either in the templating or in the incoming nucleotide, allowed to conclude that the template-binding pocket is flexible enough to allow both the *anti* and *syn* conformations [61,62]. However, extensive structural and biochemical analyses showed that the incoming nucleotide-binding pocket is much more restrictive and specific amino acids in the active site of the DNA polymerases greatly determine the specificity for the insertion of 8oxodGMP into DNA by stabilizing either the *syn* or *anti* conformation of the glycosidic bond [reviewed in [63]]. Thus, in family X DNA polymerases β and λ an asparagine residue (Asn279 in Pol β and Asn513 in Pol λ) stabilizes the incoming 8oxodGTP (*syn*) through a hydrogen bonding with the C8 carbonyl [64,65,66]. The preferential insertion of 8oxodGMP in front of dC showed in the family B DNA polymerase from bacteriophage phi29 has been hypothesized to reside in the presence of a conserved lysine residue that would impair the *syn* conformation opposite dA [67]. In family Y DNA polymerases κ and η, the preferred insertion of 8oxodGMP opposite dA depends on a Tyr and Arg residue, respectively (reviewed in [63]). Therefore, the absence of contacts between the nitrogen base and PolDom could entail an advantage as it would provide the polymerization active site of bacterial LigDs with the flexibility required to accommodate both the *anti* and *syn* conformations of the incoming 8oxo*GTP* during NHEJ, at the expense of inserting the promutagenic *syn* conformation.

The persistence of ribonucleotides into the genomic DNA poses a threat to genome stability as they can block essential processes as DNA replication and transcription, and the 2′-O can attack nucleophilically the ribonucleotide sugar backbone, destabilizing the double helix of DNA [68]. Ribonucleotides embedded in the genome are repaired by the RNase HII dependent ribonucleotide excision repair (RER) [69], wherein RNAse HII cleaves the phosphodiester bond at the 5′ side of the *NMP*, followed by removal and resynthesis by bacterial DNA polymerase I and final seal of the resulting nick by a DNA ligase [70]. As mentioned above, ROS is the main source of 8oxo*GTP* that is incorporated into DNA during DNA replication by replicative DNA polymerases, during translesion synthesis by family Y DNA polymerases and during BER by family X DNA polymerases [71,72,73,74]. The results presented here show the ability of bacterial PolDom to insert 8oxo*GTP* with an efficiency similar to that of the non-oxidized nucleotide. Therefore, it is reasonable to envision bacterial NHEJ as a source of embedded 8oxo*GMP* into the bacterial genome. In addition to the above mentioned inherent risks that entail the presence of ribonucleotides in the DNA, 8oxo*GMP* has a dual coding potential as in its *syn* conformation forms a Hoogsteen pair with adenine that can cause G to T transversion mutations if remains unrepaired. Recent studies have shown that bacterial RNase HII is able to cleave 8oxo*GMP* in both the *anti* and *syn* conformations within the DNA [75,76], suggesting that the RER pathway could prevent the detrimental effects of the 8oxo*GMP* inserted by bacterial PolDom during NHEJ.

## 4. Conclusions

Previous studies clearly established the ability of the catalytic active site of bacterial NHEJ to use preferentially *NTPs* as the substrate to perform efficient DSB repair by NHEJ during cell cycle stages where the dNTP pool is depleted, as it occurs during the stationary phase or sporulation. The results presented here show that such a preference is due principally to an absolutely conserved histidine that interacts with the 2′-OH group of the nucleotide, contributing also to the plasticity of the active site to misinsert nucleotides. Additionally, a templating nucleotide interacting lysine is of importance to stabilize/promote primer dislocation at upstream positions, an important ability shared by these enzymes to deal with noncompatible ends during the joining reaction. Finally, we show for the first time the efficiency exhibited by PolDom to use oxidized nucleotides at the expense of provoking genome instability.

## Figures and Tables

**Table 1:** Steady-state kinetic parameters of nucleotide incorporation by wild-type and mutant derivatives of *Pa*-PolDom.

Protein	Nucleotide	*k_cat_* (s^−1^)	*K_m_* (nM)	Cat.eff. (s^−1^·nM^−1^)	*f*	*F_ins_*
wild−type	UTP dTTP GTP	26.9 ± 0.8 2.2 ± 0.1 0.92 ± 0.02	58.4 ± 5.1 33.3 ± 4.9 1211 ± 90	0.46 ± 0.02 0.07 ± 0.01 8 × 10^−4^ ± 3 × 10^−5^	7	625
S768Y	UTP dTTP GTP	14.8 ± 0.4 0.42 ± 0.02 0.49 ± 0.01	55.5 ± 3.5 70.4 ± 9.9 865 ± 70	0.27 ± 0.01 6 × 10^−3^ ± 9 × 10^−4^ 6 × 10^−4^ ± 3 × 10^−5^	44	473
S768A	UTP dTTP GTP	22.6 ± 1.5 2.2 ± 0.1 0.25 ± 4 ×10^−3^	46 ± 9 31.2± 4.6 890 ± 66	0.49 ± 0.15 0.07 ± 3 × 10^−3^ 3 × 10^−4^ ± 6 × 10^−6^	7	1749
H651A	UTP dTTP GTP	6.2 ± 0.2 2.2 ± 0.2 0.02 ± 4 × 10^−4^	117 ± 10 60 ± 13 2385 ± 200	0.05 ± 4 × 10^−3^ 0.04 ± 6 × 10^−3^ 7 × 10^−6^ ± 7 ×1 0^−7^	1.3	7361

Data are means ± standard error of three independent experiments. Cat.eff. stands for catalytic efficiency: *k_cat_*/*K_m_*; *f*: (*k_cat_*/*K_m_*)*_UTP_*/(*k_cat_*/*K_m_*)_dTTP_; *F_ins_*: (*k_cat_*/*K_m_*)*_UTP_*/(*k_cat_*/*K_m_*)*_GTP._*
